# In Memory of Prof. T. G. Richardson, M.D.

**Published:** 1892-06

**Authors:** 


### IN MEMORY OF PROF. T. G. RICHARDSON, M. D.

Dr. T. G. Richardson, the veteran surgeon of New Orleans, died May 26th. The faculty of the medical department, Tulane University, of which he had so long been a member, adopted the following resolutions:

MEDICAL DEPARTMENT, TULANE UNIVERSITY<br>OF LOUISIANA,<br>NEW ORLEANS, LA., May 30, 1892.

The following resolutions were this day unanimously adopted by the faculty:

WHEREAS, Prof. T. G. Richardson, M. D., was called to New Orleans as a citizen by the medical department of the Tulane University of Louisiana, and continued his connection therewith from April 19, 1858, until severed by death, May 26, 1892, and having given to the medical department thirty-one years of active service, fourteen years as professor of anatomy, seventeen years as professor of surgery, and twenty of these years as dean; and having also given, during the last three years of retirement from active service, the most convincing proofs of his great devotion to the present and future welfare of the medical department.,

*Resolved*, That Prof. Richardson, endowed by nature with physical, mental and moral superiority, was pre-eminently distinguished for his culture and skill as surgeon and physician, which gained for him national reputation and rendered him one of the most instructive and popular of medical teachers; for exceptional scientific attainments, which, while broadening his views of nature's God, left him none the less firm in his Christian faith; for his courage and patriotism in war and his benevolence and philanthropy in peace; for his moderation and wisdom in council, and for his zeal and ability in executive administration; for his inflexible devotion to truth, honor and duty; for the strength of his friendships in adversity as in prosperity, and for the fidelity, tenderness and devotion given to his beloved and honored wife.

*Resolved*, That by the death of this strong, wise and good man the medical department has lost its most valued friend and counsellor; the medical profession its most honored representative in New Orleans; the State of Louisiana a citizen unsurpassed for patriotism and for worth; his friends a heart to love and a hand to help them, and his wife and family one who has left precious memories of a loving, virtuous and noble life.

*Resolved*, That at the next annual commencement, April 5, 1893, memorial addresses upon the life and services of Prof. T. G. Richardson, M. D., shall be delivered.

STANFORD E. CHAILLE, M. D., Dean.

---

**Our Next.**—The JULY number of the JOURNAL will begin Vol. 8; and to commemorate the beginning of our 8th year of success, we will issue a *double edition* of 2000 copies, illustrated with the portraits of the members of the Faculty of the Texas Medical College (Medical Department University of Texas.) Will take a few advertisements for that edition only. Full page $10; half page $6; one-fourth page $3.50. Extra copies 25 cts.; $1.50 per dozen, or $10 per 100.

---

**Wedding.**—The JOURNAL acknowledges the courtesy of an invitation to the wedding of Dr. H. M. Whelpley to Miss Laura Eugenie Sprannagel, to take place in St. Louis Wednesday, June 29th. Dr. Whelpley is well known throughout the South as a physician, a teacher and a journalist. The JOURNAL extends its hearty congratulations to the Doctor and his bride, and wishes them a long life of happiness.

---

**Special Notice to Manufacturers.**—Two preferred pages in the JOURNAL (between reading matter) will be for sale after this JUNE issue. Speak promptly ; it is a rare occurrence. Terms on application.

